# Persistent pancreatic enzyme elevation is correlated with superinfection in acute pancreatitis with fluid collections: a prospective, multicentre cohort analysis

**DOI:** 10.3389/fcimb.2026.1808503

**Published:** 2026-07-07

**Authors:** Balázs Lázár, Zsolt Abonyi-Tóth, Vivien Vass, Andrea Szentesi, Dalma Dobszai, Áron Vincze, Ferenc Izbéki, Mária Papp, László Czakó, Péter Jenő Hegyi, Bálint Erőss, Péter Hegyi, the Hungarian Pancreatic Study Group

**Affiliations:** 1 Institute for Translational Medicine, Medical School, University of Pécs, Pécs, Hungary; 2 Institute of Pancreatic Diseases, Semmelweis University, Budapest, Hungary; 3 Centre for Translational Medicine, Semmelweis University, Budapest, Hungary; 4 Department of Biostatistics, University of Veterinary Medicine, Budapest, Hungary; 5 Department of Gastroenterology, First Department of Medicine, Medical School, University of Pécs, Pécs, Hungary; 6 Department of Internal Medicine, Szent György Teaching Hospital of Fejér County, Székesfehérvár, Debrecen, Hungary; 7 Division of Gastroenterology, Department of Internal Medicine, Faculty of Medicine, University of Debrecen, Debrecen, Hungary; 8 Center for Gastroenterology, Department of Medicine, Albert Szent-Györgyi Medical School, University of Szeged, Szeged, Hungary; 9 Translational Pancreatology Research Group, Interdisciplinary Centre of Excellence for Research Development and Innovation, University of Szeged, Szeged, Hungary

**Keywords:** acute pancreatitis, biomarkers in acute pancreatitis, pancreatic fluid collection, persistent pancreatic enzyme elevation, risk stratification, superinfection

## Abstract

**Background:**

Superinfection of pancreatic fluid collections (PFCs) is a major determinant of late morbidity and mortality in acute pancreatitis (AP). Persistent Pancreatic Enzyme Elevation (PPEE), may contribute to fluid stasis and infection; however, its clinical impact remains incompletely defined.

**Methods:**

From 3,694 patients with AP enrolled in the Acute Pancreatitis Registry (2012–2023, 22 centres), 912 patients with PFCs were analysed. Based on pancreatic enzyme dynamics, patients were classified as having PPEE (n=276; amylase or lipase level ≥300U/l for at least one day, or mean amylase or lipase level ≥150U/l on or after day 5 of admission) or normal enzyme kinetics (NEK) (n=503). The primary outcome was PFC infection rate; secondary outcomes included mortality, organ failure, Intensive Care Unit (ICU) requirement, need for intervention, and length of hospitalisation (LOH).

**Results:**

Patients with PPEE were younger (mean age: 56.9 *vs*. 52.7 years, p<0.001) and had lower body mass index (BMI) (mean BMI: 29.3 *vs*. 26.4 kg/m^2^, p<0.001), while sex distribution was similar between groups. Alcoholic aetiology (20% *vs*. 31%, OR;1.79; CI:1.28-2.5; p<0.001), history of smoking (47.5% *vs*. 61.3%, OR: 1.76; CI: 1.3-2.38; p<0.001), and previous pancreatic disease (29.1% *vs*. 41.8%, OR: 1.75; 1.27-2.41, p<0.001) were independently associated with PPEE. Superinfection occurred more frequently in patients with PPEE compared with those with NEK (25.0% *vs*. 16.0%; OR 1.86, 95% CI 1.15–3.00; p=0.011). PPEE was also associated with a higher need for endoscopic ultrasound–guided and percutaneous interventions. In contrast, PPEE was not associated with differences in mortality, disease severity, organ failure, ICU requirement, or LOH.

**Conclusion:**

PPEE are associated with an increased risk of superinfection and the need for intervention in PFCs, without affecting disease severity or mortality in AP. Regular enzyme monitoring and early ductal assessment may enhance patient management.

## 1 Introduction

Acute pancreatitis (AP) is a common gastrointestinal disorder, classified into two main morphological forms according to the revised Atlanta classification: interstitial oedematous pancreatitis (90-95%) and necrotizing pancreatitis (5-10%) (Banks et al., 2013). The annual incidence of AP shows marked geographic variation worldwide, ranging from 4.37 to 134 per 100,000 population (Iannuzzi et al., 2022). Although approximately 80% of patients experience a mild disease course, around 20% develop moderately severe or severe AP, characterised by persistent organ failure and/or local complications such as pancreatic necrosis and peripancreatic fluid collections, with a substantially increased risk of mortality in these subgroups (Banks et al., 2013; Metri et al., 2024). Among the early complications, multi-organ failure represents the leading cause of death. In contrast, late mortality is predominantly driven by septic complications, most commonly resulting from infected peripancreatic fluid collections or superinfection of pancreatic necrosis (Mutinga et al., 2000; Liu et al., 2024). Owing to advances in supportive and interventional management, the overall mortality of AP has declined to approximately 2% in recent decades. However, mortality remains around 3% in moderately severe AP and reaches approximately 20% in severe cases (Mihoc et al., 2024). During the late phase of the disease, AP is frequently complicated by pancreatic pseudocysts and walled-off necrosis (WON). Secondary superinfection of these collections is associated with markedly increased morbidity and mortality and continues to pose a major challenge in clinical management (Petrov et al., 2010). While the overall mortality of AP is reported to be between 1% and 5%, it can rise to 11-20% in severe and complicated cases. Mortality has been shown to be significantly higher in patients with superinfected necrosis (35.2%) compared with those with organ failure and sterile necrosis (19.2%). Pancreatic fluid collections (PFCs) occur in approximately 42.7% of patients, whereas the long-term incidence of pseudocyst formation is considerably lower, around 6.3%. Importantly, most fluid collections resolve spontaneously within one month (Cui et al., 2014). These observations consistently demonstrate that mortality increases substantially in the presence of organ failure and, in particular, superinfection of pancreatic fluid collections (Gloor et al., 2001; Werge et al., 2016). Superinfection of pancreatic fluid collections is therefore considered one of the most critical determinants of late morbidity and mortality in AP. Contemporary analyses consistently indicate that superinfection - rather than the extent of necrosis alone - drives adverse clinical outcomes, prolonged hospitalisation, and the need for invasive interventions (Párniczky et al., 2019; Tarján et al., 2024; Cai et al., 2025). Emerging clinical evidence and mechanistic studies suggest that the obstructed pancreatic duct plays a pivotal role in the development of pancreatic fluid collections and their subsequent risk of superinfection (Pallagi et al., 2015). In other organ systems, fluid retention is a well-established predisposing factor for superinfection as exemplified by cholangitis (House et al., 2006; Goldberg et al., 2013). Biochemical markers of pancreatic juice stasis may include persistently elevated serum amylase and lipase levels, which can occur with obstructed pancreatic ducts during pancreatitis (Chen et al., 2000).

The aim of the present study is to determine the incidence of pancreatic fluid collections in a prospective cohort of patients with AP and to investigate the association between superinfection of these collections and persistent pancreatic enzyme elevation (PPEE) we examined.

## 2 Methods

### 2.1 Study design and settings

We performed a *post-hoc* analysis using clinical data derived from the prospective, multicentre Acute Pancreatitis Registry of the Hungarian Pancreatic Study Group (ethical approval numbers: 22254-1/2012/EKU, 36305-1/2016/EKU, 17787-8/2020/EÜIG). The study was conducted in accordance with the STROBE guidelines for cohort studies (von Elm et al., 2007) (**Supplementary Figure 1**).

The Acute Pancreatitis Registry has previously served as the basis for several high-quality registry-based analyses addressing disease severity, complications, and outcomes, including detailed characterization of post-discharge mortality (Czapári et al., 2023; Gieszinger et al., 2025; Hussein et al., 2025; Lee et al., 2025; Pázmány et al., 2025; Szentesi et al., 2025). Data were prospectively collected from a nationwide registry comprising patients diagnosed with AP across 22 centres in Hungary between 2012 and September 2023. Long-term follow-up information was obtained through the national electronic health services system and from the Hungarian Ministry of the Interior.

### 2.2 Participants and definitions

Inclusion criteria comprised patients aged 0–99 years with a diagnosis of AP and admitted to participating centres (**Supplementary Table 2**), whose legal guardians or, in the case of adults (≥18 years), the patients themselves provided informed consent for inclusion in the registry. Missing data for the variables analysed are detailed in the Results section.

Patients were diagnosed with AP based on the presence of at least two of the following three criteria: characteristic abdominal pain, elevation of pancreatic enzymes to at least three times the upper limit of normal, and/or imaging findings consistent with AP according to the revised Atlanta classification (Banks et al., 2013). From the overall registry population (n = 3,694), 912 patients who developed early or late inflammatory fluid collections were identified. Of these, 779 patients had sufficient pancreatic enzyme data (amylase and/or lipase) to allow appropriate grouping. Because imaging data on ductal obstruction (e.g., ductal dilation, strictures, or disconnected pancreatic duct syndrome) were not uniformly available, we classified patients based solely on biochemical criteria. To avoid implying radiologically confirmed obstruction, the term persistent pancreatic enzyme elevation (PPEE), and the comparison group is normal enzyme kinetics (NEK).

The exposure group consisted of 276 patients who exhibited persistent pancreatic enzyme elevation (PPEE) of at least one pancreatic enzyme on day 5 or later after admission (amylase or lipase ≥300 U/l for at least one day or mean amylase or lipase ≥150 U/l). The control group consisted of 503 patients who showed normal enzyme kinetics (NEK) after day 5. (**Supplementary Figure 2**). Definitions, disease severity, and clinical endpoints were assessed in accordance with the most recent international guidelines for AP (International Association of Pancreatology Revised Guidelines on Acute Pancreatitis 2025: Supported and Endorsed by the American Pancreatic Association, European Pancreatic Club, Indian Pancreas Club, and Japan Pancreas Society, 2025). A total of 133 patients (14.6%) with local complications could not be classified into PPEE or NEK groups due to insufficient longitudinal enzyme data (fewer than 5 days of amylase/lipase measurements). Missingness resulted from irregular or delayed blood sampling, early mortality preventing adequate follow-up, inter-institutional transfers, or centre-level variability in laboratory protocols. Baseline characteristics of these patients (age, sex, BMI, aetiology, severity, organ failure, mortality) were comparable to those with complete data, indicating that missingness was random with respect to clinical outcomes. (**Supplementary Table 3**). These patients were excluded only from PPEE/NEK classification but were retained in all descriptive analyses.

### 2.3 Variables, data collection, and outcome parameters

Local complications were defined according to the revised Atlanta classification (Banks et al., 2013), based on timing (early *vs*. late) and the presence or absence of necrosis, in patients with available pancreatic enzyme measurements (n=779) (**Supplementary Figure 3**). The distribution of local complications was as follows:

- Early complications (before 4 weeks):
  - Acute peripancreatic fluid collection (n=308, 39.54%).
  - Acute necrotic collection (n=208, 26.7%).
- Late complications (after 4 weeks):
  - Pancreatic pseudocyst (n=176, 22.59%).
  - Walled-off necrosis (n=87, 11.17%).

PPEE was defined as an elevation of at least one pancreatic enzyme from day 5 after symptom onset, characterized by amylase ≥300 U/L (≥3× ULN; ULN 100 U/L) or lipase ≥300U/L (≥5× ULN; ULN 60 U/L) on at least one day, or a mean amylase or lipase ≥150 U/L on subsequent days. NEK was defined by enzyme levels remaining below 3× ULN for amylase and 5x ULN for lipase from day 5 onward, with mean values on subsequent days not exceeding 150 U/l. These thresholds were established based on previously published data describing the normalization kinetics of pancreatic enzymes following AP. Prior studies indicate that serum amylase levels typically normalize by day 5 after onset; therefore, a cut-off of 3x ULN was applied. In contrast, lipase levels may remain elevated for 8–14 days, and a 1.5-2x ULN elevation can still be observed on day 5, even in cases of average pancreatitis. Accordingly, a higher threshold of 5x ULN was selected for lipase to preserve diagnostic sensitivity (Yadav et al., 2002; Jasdanwala and Babyatsky, 2015; Tenner et al., 2024). Pancreatic enzyme kinetics were evaluated from the day of hospital admission. Because symptom onset timing was inconsistently documented, all temporal references (e.g., ‘day 5’) refer to hospitalization days rather than days from symptom onset.

Patients were categorized into the following infection-related groups:

- Verified pancreatic fluid superinfection: confirmed superinfection based on puncture results or imaging findings (CT or MRI).
- Suspected pancreatic fluid superinfection: absence of alternative infectious sources, accompanied by a compatible clinical picture (procalcitonin >1 ng/mL, fever >38°C, elevated inflammatory markers responding exclusively to antibiotic therapy).
- Other infection: documented non-pancreatic infections (e.g., pneumonia, cholecystitis, cholangitis, Clostridium difficile infection, urinary tract infection, etc.).
- No infection: absence of clinical or laboratory signs of infection.
- Symptomatic cysts without superinfection: absence of infection, but presence of local complications related to fluid collections (e.g., gastric outlet syndrome, vascular compression, biliary obstruction).

The primary outcome of the study was the rate of infection of pancreatic fluid collections in NEK and PPEE groups. Secondary outcomes included 1-year mortality, disease severity, the association between superinfection and aetiology, length of hospitalisation (LOH), Intensive Care Unit (ICU) requirement, and the need for interventional procedures in NEK and PPEE groups.

### 2.4 Data collection

Patients’ clinical parameters were recorded prospectively; however, data regarding superinfection of local effusions were analysed retrospectively based on detailed clinical review and consensus assessment by two experienced pancreatologists. Patients were subsequently classified into predefined subgroups: verified cyst infection, suspected cyst infection, other infection, no infection, and symptomatic cyst. A minimum follow-up duration of 1 year was available for 3,004 patients to assess the main study endpoints. Complete follow-up could not be obtained for all patients due to loss to subsequent contact.

### 2.5 Statistical methods

Descriptive statistics were used to characterize and compare the baseline cohort, the total registry population, and the analysed study cohort. Categorical variables are presented as proportions, while continuous variables are reported as means with standard deviations (SD). Binomial logistic regression was applied to model the association between explanatory variables and study outcomes. Univariable logistic regression models were constructed by including only one explanatory variable at a time. Odds ratios (ORs) with corresponding 95% confidence intervals (CIs) were reported. Graphical representations display both observed and predicted risks; for categorical variables, these values are identical. As univariable analyses do not account for potential confounding, results were interpreted with caution. Risk factor analyses for PPEE were performed using univariable models, as the objective was descriptive. Variables such as necrosis and severity were not included as covariates because they may represent downstream consequences of impaired pancreatic duct.

Multivariable binomial logistic regression models were subsequently used to assess the association between PPEE and study outcomes while adjusting for potential confounders. All multivariable models were adjusted for age and sex. In analyses of aetiology, the combined alcohol + hypertriglyceridemia category was merged with the mixed aetiology group. Multivariable models were adjusted for age and sex. Additional variables were not included to avoid overfitting and because several (e.g., BMI, aetiology, smoking, necrosis, severity) may represent downstream effects of impaired pancreatic duct. All regression analyses were performed using R software version 4.4.2, employing the emmeans package (version 1.10.6) and partykit package (version 1.2-23) where appropriate. A Classification and Regression Tree (CART) approach was applied to identify predictors of the outcome. The model was constructed using recursive binary partitioning, selecting splits that maximized within-node homogeneity.

## 3 Results

### 3.1 General characteristics of the analysed population

A total of 3,964 patients were included in the analysis. Among this cohort, 912 patients developed pancreatic fluid collection-related complications following AP and were included in the present analysis (**Table 1**).

**Table 1:** Basic characteristics of the analysed cohort.

Variables	Analysed cohort	No local complication group	Local complication group	p-value (Analysed vs. Local comp.)	Test
**Number of cases**	**3,694**	**2,782**	**912**		
Sex					
**Male (%)**	2,103 (56.93%)	1,489 (53.52%)	614 (67.32%)	**<0.001**	X^2^
**Female (%)**	1,591 (43.07%)	1,293 (46.48%)	298 (32.68%)		
**Age - years (median, IQR)**	58 (44-71)	59 (44-72)	55 (43-67)	**0.012**	M-W U
**BMI (median, IQR)**	27.18 (23.67-31.48)	27.14 (23.59-31.39)	27.54 (24.13-31.64)	0.42	M-W U
Risk factors					
**Alcohol consumption**	1,701/3,582 (47.49%)	1,207/2,690 (44.87%)	494/892 (55.38%)	**<0.001**	X^2^
**Smoking previously**	1,723/3,565(48.33%)	1,247/2,679 (46.55%)	476/886 (53.72%)	**0.003**	
Etiology					
**Hypertriglyceridemia**	139/3,694 (3.76%)	73/2,782 (2.62%)	66/912 (7.24%)	**<0.001**	X^2^
**Biliary**	1,462/3,694 (39.58%)	1,249/2,782 (44.9%)	213/912 (23.36%)	**<0.001**	
**Alcoholic**	665/3,694 (18.00%)	435/2,782 (15.64%)	230/912 (25.22%)	**<0.001**	
**Idiopathic**	669/3,694 (18.11%)	490/2,782 (17.61%)	179/912 (19.63%)	0.29	
**Post-ERCP**	118/3,694 (3.19%)	98/2,782 (3.52%)	20/912 (2.19%)	0.18	
**Alcoholic+HTG**	73/3,694 (1.98%)	34/2,782 (1.22%)	39/912 (4.28%)	**<0.001**	
**Mixed**	75/3,694 (2.03%)	46/2,782 (1.65%)	29/912 (3.18%)	0.09	
**Other**	493/3,694 (13.35%)	357/2,782 (12.83%)	136/912 (14.91%)	0.11	
Severity					
**Mild**	2,503/3,683 (67.96%)	2,503/2,774 (90.23%)	0/909 (0%)	**<0.001**	X^2^
**Moderate**	927/3,683 (25.17%)	171/2,774 (6.16%)	756/909 (83.17%)	**<0.001**	
**Severe**	253/3,683 (6.87%)	100/2,774 (3.6%)	153/909 (16.83%)	**<0.001**	
**Mortality 1 year follow up**	275/3,004 (9.15%)	157/2,250 (6.98%)	118/754 (15.65%)	**<0.001**	X^2^
**In-hospital mortality**	110/3,660 (3.01%)	43/2,754 (1.56%)	67/906 (7.4%)	**<0.001**	X^2^
**LOH (median, IQR)**	8 (6–12)	7 (5–9)	13 (8–19)	**<0.001**	M-W U
**ICU needed at admission**	151/3,350 (4.51%)	80/2,524 (3.17%)	71/826 (8.6%)	**<0.001**	X^2^
**Antibiotic usage**	2,282/3,522 (64.79%)	1,600/2,649 (60.4%)	682/873 (78.12%)	**<0.001**	X^2^
**Previous pancreatic disease**	1,121/3,514 (31.90%)	830/2,673 (31.05%)	291/841 (34.6%)	0.07	X^2^
**Organ failure during hospital stay**	424/3,689 (11.49%)	137/2,590 (5.29%)	217/911 (23.82%)	**<0.001**	X^2^

IQR, interquartile range; BMI, body mass index; ERCP, endoscopic retrograde cholangiopancreatography; LOH, length of hospitalisation; ICU, intensive care unit; X^2^, Pearson’s Chi-square test; M-W U, Mann-Whitney U test. A significant difference at a p-value below 0.05

Bold values indicate statistically significant differences between the compared groups. These values highlight variables where the p‑value is below the significance threshold (p < 0.05), based on the statistical tests applied (Pearson’s Chi‑square test or Mann–Whitney U test).

### 3.2 Risk factors of PPEE

**Figure 1:**
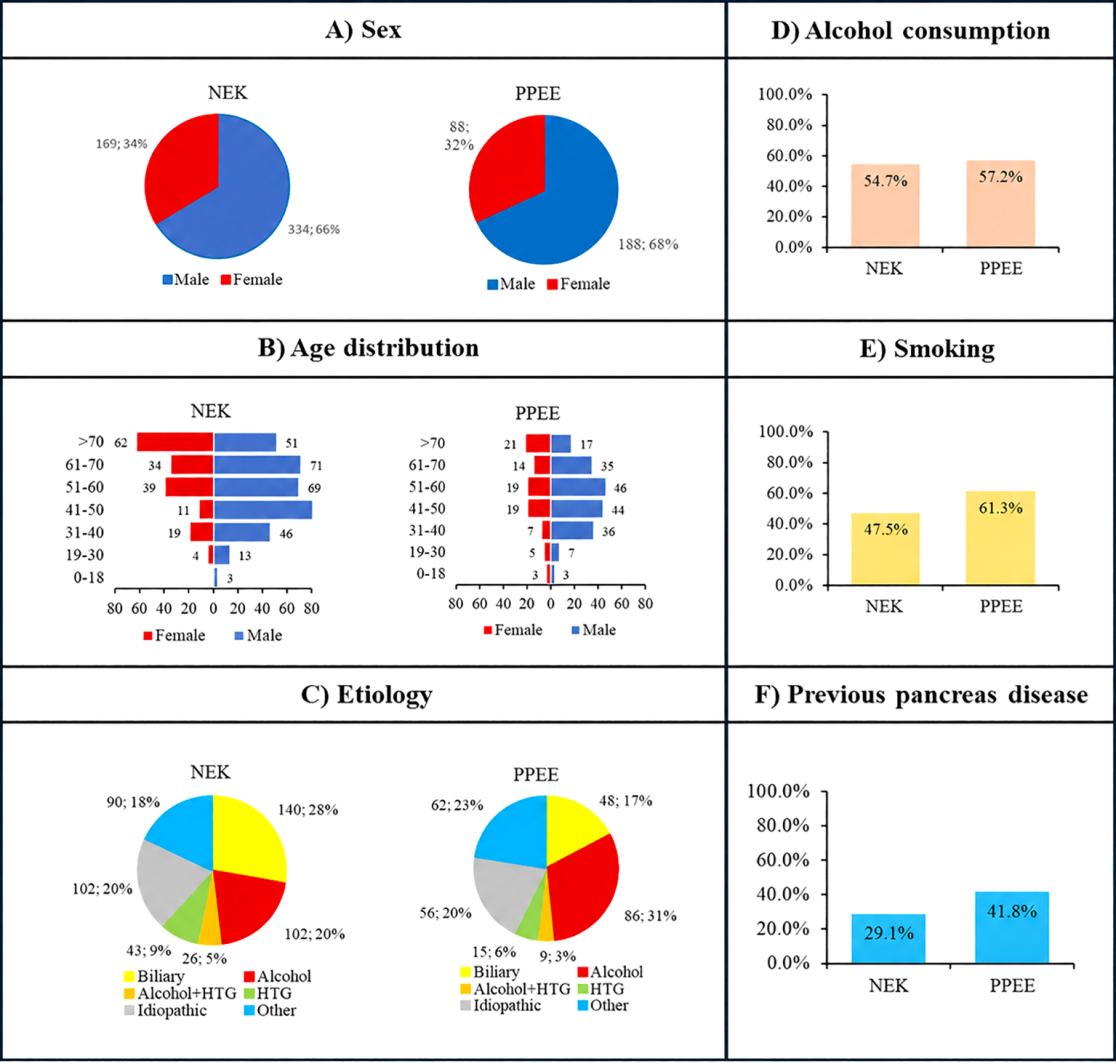
Risk factors of persistent pancreatic enzyme elevation. **(A)** Sex **(B)** Age distribution. **(C)** Aetiology. **(D)** Alcohol consumption. **(E)** Smoking. **(F)** Previous pancreatic disease. NEK: normal enzyme kinetics, PPEE: persistent pancreatic enzyme elevation, IQR: interquartile range, HTG: hypertriglyceridemia, ERCP: endoscopic-retrograde-cholangiopancreatography, AP: acute pancreatitis.

Among the 912 patients with local complications following AP (24.6% of the total cohort), 503 patients (55.1%) had NEK, while 276 patients (30.3%) were classified as having PPEE. Pancreatic enzyme data were insufficient for classification in 133 patients (14.6%). Patients with PPEE were significantly younger than those with NEK (mean age: 56.9 *vs*. 52.7, p<0.001), although the overall age distribution patterns were similar between the two groups (**Figure 1B****).** Body mass index (BMI) was also significantly lower in the PPEE group compared with the NEK group (mean BMI: 28.71 *vs*. 25.73, p<0.001) (**Supplementary Table 3**). These differences may contribute to secondary outcomes. The sex distribution did not differ significantly between groups (p=0.59) (**Figure 1A**). Alcoholic aetiology (20% *vs*. 31%, OR;1.79; CI:1.28-2.5; p<0.001) (**Figure 1C****),** history of smoking (47.5% *vs*. 61.3%, OR: 1.76; CI: 1.3-2.38; p<0.001) (**Figure 1E****),** and previous pancreatic disease (29.1% *vs*. 41.8%, OR: 1.75; 1.27-2.41, p<0.001) (**Figure 1F**) were identified as risk factors for PPEE. In contrast, overall alcohol consumption did not differ significantly between the groups (54.7% *vs*. 57.2%, p=0.53) (**Figure 1D****).**

### 3.3 Infection of pancreatic fluid collections

**Figure 2:**
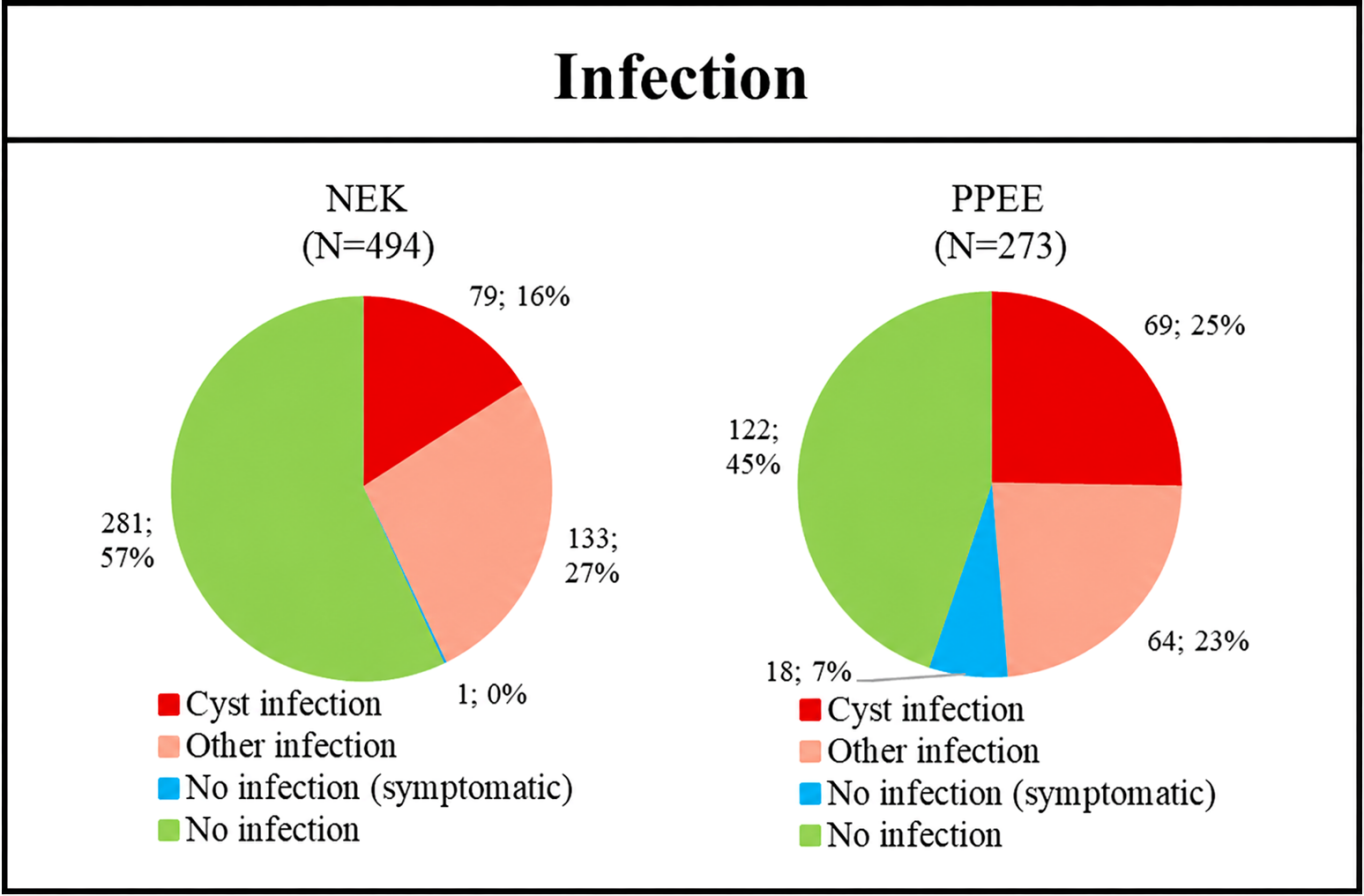
**(A)** Superinfection rate in pancreatic fluid collections, NEK, normal enzyme kinetics; PPEE, persistent pancreatic enzyme elevation.

Among patients with infected pancreatic fluid collections, verified cyst superinfection was identified in 38.92% (65/167), whereas suspected cyst superinfection accounted for 61.08% (102/167). In multivariable analysis, superinfection of pancreatic fluid collections occurred significantly more frequently in patients with PPEE than in those with NEK (25.3% *vs* 16%; OR, 1.86; CI, 1.15-3.0; p = 0.011) (**Figure 2**). PPEE was therefore strongly associated with an increased risk of post-pancreatitis fluid collection superinfection. Clinical symptoms attributable to pancreatic fluid retention were also observed at a significantly higher rate in patients with persistently elevated amylase or lipase levels compared with those with normal enzyme profiles (0.2% *vs*. 6.6%, OR: 35.46, CI: 4.69 – 268, p<0.001) (**Figure 2**). The distribution of PFC subtypes did not differ significantly between groups. Subgroup analyses demonstrated that PPEE was associated with higher infection rates within each PFC subtype, indicating that the observed association was not driven by differences in PFC composition. (**Supplementary Figure 3**).

### 3.4 Further outcome parameters in the local complication population

**Figure 3:**
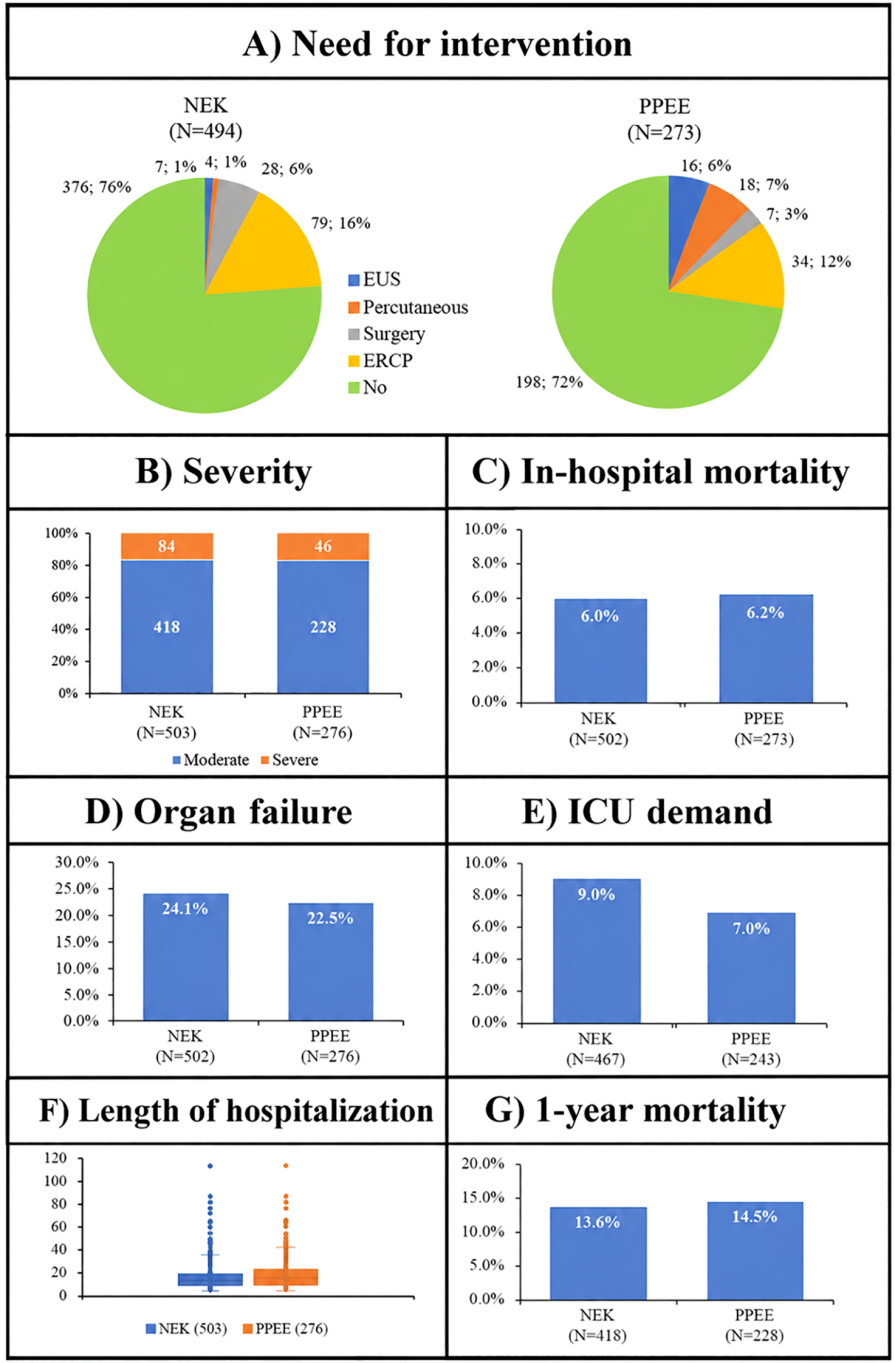
Secondary outcomes. **(A)** Need for intervention. **(B)** Severity. **(C)** In-hospital mortality. **(D)** Organ failure. **(E)** ICU demand. **(F)** Length of hospital stay. **(G)** 1-year mortality.

LOH was significantly longer in patients with local complications compared with those without such complications (median: 13 *vs*. 7 days, p<0.001) (**Table 1**). However, LOH did not differ significantly between patients with PPEE and those with NEK (17.62 *vs*. 19.25 days, p=0.12) (**Figure 3F****).** In univariate analysis, the need for EUS-guided endoscopic intervention was significantly higher in patients with PPEE compared with those with NEK (6% *vs*. 1%; OR: 5.07; 95% CI: 1.89-13.65; p = 0.0013). Similarly, percutaneous interventions were more frequently required in the PPEE group (7% *vs*. 1%; OR: 11.28; CI: 3.2-39.8; p<0.001). (**Figure 3A**). In contrast, the rate of surgical intervention did not differ significantly between groups (3% *vs*. 6%, OR:0.44, CI: 0.1-1.3, p=0.14). During the study period, EUS-guided cyst drainage was not yet widely implemented in Hungary and was mainly performed in tertiary centres and in severe cases. Consequently, these intervention rates may differ from those reported in contemporary international cohorts. PPEE was not associated with disease severity (OR: 1.14, CI: 0.71-1.83, p=0.59), 1-year mortality (14.5% *vs*. 13.6%, OR: 1.38, CI: 0.82-2.32, p=0.22), organ failure (22.5% *vs*. 24.1%, OR: 0.72, CI: 0.41-1.26, p=0.25), or ICU requirement (7% *vs*. 9%, OR: 0.83, CI: 0.45-1.51, P = 0.53) (**Figures 3B–E, G****).**

### 3.5 Further analyses

The pancreatic fluid collection group was further classified as early or late and associated with oedematous or necrotizing pancreatitis, in accordance with the revised Atlanta classification (Banks et al., 2013). Late fluid collections were associated with a significantly higher rate of superinfection compared with early collections (32.32% *vs*. 12.2%, OR: 3.45, CI: 2.4-4.9, p<0.001). Similarly, the proportion of symptomatic cases was markedly higher among late fluid collections (6.46% *vs*. 0.4%, OR: 17.9, CI: 4.2-76.9, p<0.001). In contrast, mortality did not differ significantly between early and late fluid collections (13.69% *vs*. 10.5%, OR: 1.36, CI: 0.89-2.1, p=0.13) (**Supplementary Figure 3**). Additional analyses exploring factors associated with clinical outcomes are presented in **Supplementary Table 4**.

## 4 Discussion

In this multicentre registry-based analysis, we evaluated 779 patients with AP who had sufficient pancreatic enzyme data and detailed information on PFCs. Persistently elevated serum amylase or lipase levels beyond day 5 of AP hospitalization were used as a surrogate marker of impaired pancreatic duct. Our findings demonstrate that patients with PPEE had a significantly higher rate of superinfection compared with those with NEK. The observed absolute increase of 9.3% in infection rate suggests that sustained pancreatic enzyme elevation may reflect unresolved ductal obstructions and pancreatic juice stasis, conditions that are biologically favourable for bacterial translocation and secondary infection. Consistent with this interpretation, patients with PPEE required significantly more endoscopic and percutaneous interventions, reflecting a higher burden of clinically relevant local complications. Importantly, PPEE were not associated with increased rates of organ failure, LOH, surgical intervention, ICU requirement, or in‐hospital and 1‐year mortality. These findings indicate that while PPEE primarily influences late local infectious complications and intervention needs, it does not appear to drive systemic disease severity or overall mortality in this cohort. Alcoholic aetiology, a history of smoking, previous pancreatic disease, and younger age were identified as significant risk factors for PPEE. Together, these associations support a pathophysiological link between pre-existing pancreatic vulnerability, impaired ductal clearance, and the development of infected pancreatic fluid collections.

### 4.1 Infection

Current medical literature provides little evidence that pancreatic enzyme levels (amylase or lipase) are directly associated with the risk or presence of superinfection in post-pancreatitis pancreatic fluid collections. Only limited data have addressed the relationship between persistent elevation of pancreatic enzymes beyond the early phase of AP and subsequent infection of fluid collections. Available evidence suggests that serum lipase tends to be higher in patients who later develop infected pancreatic necrosis; however, this association appears to be nonspecific. Previous meta-analyses and cohort studies indicate that inflammatory markers such as C-reactive protein (CRP) and procalcitonin (PCT), as well as overall clinical severity, are more reliable predictors of infection than serum pancreatic enzyme levels alone. In contrast, amylase has not been consistently identified as a predictive marker for infectious complications (Li et al., 2022; Tarján et al., 2024). Importantly, persistent hyperamylasemia has been linked to local complications such as pancreatic necrosis and pseudocyst formation, higher CT severity, and recurrent pancreatitis, but not specifically to infection (Kim et al., 2020). From a biological perspective, the increased risk of superinfection is thought to be driven by pancreatic juice stasis and fluid retention, which facilitate bacterial translocation – an established mechanism also observed in other organ systems, such as the biliary tract. Reduced intestinal perfusion during AP further compromises gut barrier integrity, promoting translocation of intestinal bacteria (Runkel et al., 1991; House et al., 2006). The development of superinfection is often due to decreased intestinal circulation and the subsequent easier bacterial translocation from the intestine (Runkel et al., 1991). Persistent ductal hypertension may exacerbate this process by impairing pancreatic microcirculation and enabling bacterial spread through lymphatic and vascular pathways (Pallagi et al., 2015). In the present study, we applied a distinct conceptual framework by using persistent elevation of serum pancreatic enzymes as a surrogate marker of impaired pancreatic duct rather than as a direct indicator of infection. Within this context, we observed a significantly higher rate of superinfection among patients with PPEE compared with those with NEK, consistent with previously reported infection rates in necrotic pancreatic collections (Muthusamy et al., 2016). These findings support the hypothesis that impaired ductal clearance and pancreatic juice stasis – rather than enzyme elevation alone – play a central role in the development of infected pancreatic fluid collections. PPEE may serve as an early biochemical signal of impaired ductal clearance and increased risk of PFC infection. While not diagnostic on its own, it may support decisions regarding earlier imaging, closer monitoring, or timely referral to centres with interventional expertise. In most cases, enzyme elevation preceded infection diagnosis; however, due to retrospective adjudication, reverse causation cannot be excluded”.

### 4.2 Predisposing factors for PPEE

Previous studies have identified several factors predisposing to pancreatic ductal stasis and the development of post-pancreatitis fluid collections, including pancreatic duct disruption, necrotizing pancreatitis, alcohol-related aaetiology, younger age, elevated early inflammatory markers, delayed hospital admission, and higher computed tomography severity index scores (Cui et al., 2014; Muthusamy et al., 2016; Manrai et al., 2018). These factors are thought to reflect increased pancreatic vulnerability and impaired clearance of pancreatic secretions. Pancreatic ductal secretion is essential for maintaining adequate intraductal flow and preventing pancreatic juice stasis. This process is tightly regulated by the cystic fibrosis transmembrane conductance regulator (CFTR), which controls bicarbonate and chloride transport in pancreatic ductal epithelial cells. Impaired CFTR function results in reduced ductal fluid and bicarbonate secretion, increased viscosity of pancreatic juice, and elevated intraductal pressure, thereby predisposing to ductal obstruction and fluid retention. Beyond classical cystic fibrosis, heterozygous or mild CFTR mutations, as well as functional CFTR impairment, have been robustly associated with pancreatic ductal dysfunction, recurrent pancreatitis, and delayed clearance of pancreatic secretions. Although these alterations may not lead to overt exocrine insufficiency, they can substantially disturb ductal flow dynamics during and after episodes of AP. Consequently, CFTR-related ductal secretory dysfunction represents a biologically plausible mechanism contributing to persistent impairment of the pancreatic duct, the formation of pancreatic fluid collections, and their subsequent complications (Hegyi et al., 2016; Pallagi et al., 2024; Venglovecz et al., 2024). In our cohort, younger age, lower BMI, alcoholic aaetiology, history of smoking, and previous pancreatic disease emerged as independent predisposing factors for PPEE. Earlier studies have similarly linked alcohol-related pancreatitis and early-stage chronic pancreatic injury to younger patient populations, with smoking acting as an important modifying risk factor (Yadav and Lowenfels, 2013; Forsmark et al., 2016). Although chronic pancreatitis itself was not directly assessed in the present study, alcohol consumption and smoking—both strongly associated with chronic pancreatic damage—were frequent among patients with PPEE. Previous reports have also demonstrated a higher prevalence of alcohol-related aaetiology in patients with disconnected pancreatic duct syndrome, further supporting the role of ductal disruption and impaired outflow in the pathogenesis of persistent fluid collections (Bang et al., 2018).

### 4.3 Clinical outcome with PPEE

Although elevated serum amylase and lipase levels are essential for the diagnosis of AP, their prognostic value is limited compared with clinical scoring systems and imaging-based classifications. In most cases of AP, serum amylase levels normalise within 3–7 days, whereas lipase levels remain elevated and return to normal within 8–14 days (Yadav et al., 2002; Tenner et al., 2024). PPEE may arise from primary mechanical obstruction, secondary inflammation-related ductal narrowing, or functional disturbances such as transient sphincter dysfunction or oedema (Delhaye et al., 2008). However, multiple studies and international consensus statements have consistently shown that serum pancreatic enzyme levels do not independently predict disease severity or mortality in AP (Lankisch et al., 1999; Yadav et al., 2002; Tenner et al., 2024; Pázmány et al., 2025). In line with these observations, we found no association between persistently elevated pancreatic enzyme levels and systemic outcomes, including disease severity, organ failure, ICU requirement, or short- and long-term mortality. Currently, no single laboratory marker reliably predicts severe AP, and established predictors include parameters reflecting haemoconcentration, systemic inflammation, and tissue hypoperfusion rather than pancreatic enzyme activity (Brown et al., 2007; Tenner et al., 2013; Yang et al., 2014; Koutroumpakis et al., 2015; Staubli et al., 2015; Valverde-López et al., 2017). Obesity and metabolic factors have also been identified as important contributors to severe disease and complications in previous meta-analyses (Dobszai et al., 2025). In our cohort, severity and 1-year mortality were primarily driven by demographic factors and previous pancreatic disease rather than by pancreatic enzyme dynamics (**Supplementary Table 4**).

### 4.4 Persistent pancreatic enzyme elevation and need for intervention in post-pancreatitis fluid collections

Most pancreatic fluid collections resolve spontaneously without intervention, and only a subset of patients require endoscopic, percutaneous, or surgical treatment (Petrov et al., 2010). In the present study, the majority of patients in both the PPEE and NEK groups were managed conservatively. Nevertheless, patients with PPEE more frequently required minimally invasive interventions, particularly endoscopic and percutaneous procedures. PPEE beyond the acute phase has previously been associated with pancreatic duct disruption, disconnected pancreatic duct syndrome, and impaired ductal clearance, all of which predispose to the persistence and complication of fluid collections (Bang et al., 2018; Timmerhuis et al., 2023; Pázmány et al., 2025). Our findings extend this concept by demonstrating that persistently elevated enzyme levels identify patients at increased risk for local infectious complications rather than systemic deterioration. This observation is consistent with current guideline recommendations, which emphasise targeted intervention in infected or obstructed necrotising pancreatitis while discouraging unnecessary early procedures (International Association of Pancreatology Revised Guidelines on Acute Pancreatitis 2025: Supported and Endorsed by the American Pancreatic Association, European Pancreatic Club, Indian Pancreas Club, and Japan Pancreas Society, 2025).

### 4.5 Strengths and limitations

This study leveraged a large, prospective, multicentre registry spanning more than a decade, encompassing a broad spectrum of AP phenotypes and enhancing the generalisability of our findings. Rigorous prospective data collection and structured follow-up through national electronic health records enabled reliable assessment of clinical outcomes, interventions, and mortality, with an overall data quality exceeding 90% (**Supplementary Table 5**).

Several limitations should be acknowledged. Classification of pancreatic fluid collection superinfection relied on retrospective chart review and expert consensus, introducing the potential for misclassification despite adjudication procedures. Pancreatic enzyme data were incomplete for a subset of patients, potentially introducing selection bias toward individuals with more intensive laboratory monitoring. Variability in documentation practices and clinical management across centres may also have influenced outcome assessment. Additionally, the timing of symptom onset relative to hospital admission was not uniformly recorded, complicating the interpretation of enzyme trajectories. Finally, as an observational registry-based study, causal inference cannot be established, and residual confounding cannot be excluded. As a *post hoc* observational analysis, causal inference is limited. Residual confounding, indirect biochemical definition of PPEE, and lack of mechanistic confirmation (e.g., ductal imaging) restrict causal interpretation. Findings should be considered hypothesis-generating. Additional limitations include the indirect biochemical definition of PPEE, heterogeneity in PFC types, and absence of uniform imaging to confirm ductal obstruction. These factors may contribute to misclassification and should be addressed in future prospective studies.

### 4.6 Implication for practice and research

After an episode of AP, periodic monitoring of pancreatic enzyme levels may help identify patients at increased risk of developing superinfection or symptomatic pancreatic fluid collections, warranting closer clinical observation. While persistent enzyme elevation should not be interpreted as a marker of disease severity, it may serve as an indicator of local risk. To better define the clinical relevance of persistent enzyme elevation, future prospective studies should incorporate standardized, protocol-based assessment of pancreatic enzyme trajectories alongside objective markers of infection and clinical outcomes. In addition, randomized trials evaluating early or symptom-guided interventions in patients with persistently elevated amylase levels may help clarify whether targeted management strategies can reduce local infectious complications without increasing unnecessary interventions (Hegyi et al., 2021; Hegyi and Varró, 2024; Hegyi and Garami, 2025).

## 5 Conclusion

PPEE is associated with an increased risk of superinfection in PFCs after AP. PPEE likely reflect impaired ductal clearance rather than disease severity and do not affect mortality or major systemic outcomes. However, closer monitoring of these patients may support earlier detection of infection and timely management.

## Data Availability

The raw data supporting the conclusions of this article will be made available by the authors, without undue reservation.
