# Supplementary material for: Persistent pancreatic enzyme elevation is correlated with superinfection in acute pancreatitis with fluid collections: a prospective, multicentre cohort analysis
Source: Front Cell Infect Microbiol. 2026 Jul 7;16:1808503. doi: 10.3389/fcimb.2026.1808503 (PMC13384862; doi:10.3389/fcimb.2026.1808503)
Supplement: Supplementary file 1 [file DataSheet1.pdf]

Persistent Pancreatic Enzyme Elevation is Correlated with Superinfection in Acute Pancreatitis with Fluid Collections: A Prospective, Multicentre Cohort Analysis

1 AUTHORS:

Balázs Lázár[1,2,3], Zsolt Abonyi-Tóth[4,5], Vivien Vass[1], Andrea Szentesi[1,4], Dalma Dobszai[1,4], Áron Vincze[6], Ferenc Izbéki[7], Mária Papp[8], László Czakó[9], Péter Jenő Hegyi[2,4], Bálint Erőss[1,2,4], Péter Hegyi[1,2,4,10] and Hungarian Pancreatic study group[11]

[1] Institute for Translational Medicine, Medical School, University of Pécs, Pécs, Hungary

[2] Institute of Pancreatic Diseases, Semmelweis University, Budapest, Hungary

[3] Semmelweis University, Department of Surgery, Transplantation and Gastroenterology

[4] Centre for Translational Medicine, Semmelweis University, Budapest, Hungary

[5] Department of Biostatistics, University of Veterinary Medicine, Budapest, Hungary

[6] Department of Gastroenterology, First Department of Medicine, Medical School, University of Pécs, Pécs, Hungary

[7] Szent György Teaching Hospital of Fejér County, Székesfehérvár, Hungary

[8] Division of Gastroenterology, Department of Internal Medicine, Faculty of Medicine, University of Debrecen, Hungary.

[9] Center for Gastroenterology, Department of Medicine, Albert Szent-Györgyi Medical School, University of Szeged, Szeged, Hungary

[10] Translational Pancreatology Research Group, Interdisciplinary Centre of Excellence for Research Development and Innovation, University of Szeged, Szeged, Hungary

[11] full names and affiliations are detailed in the (Supplementary file Table 1)

2 Table of contents:

Supplementary Table 1. Further contributors of the Hungarian Pancreatic Study Group 3-6

Supplementary Table 2. Centre distribution of the analysed cohort………………………. 7

Supplementary Table 3. Characteristics of the NEK and PPEE groups.…….……………. 8

Supplementary Table 4. Factors influencing outcomes; severity by age and BMI ……… 9-10

Supplementary Table 5. Data quality of the Hungarian Acute Pancreatitis Registry……... 11

Supplementary Table 6. Representativity of the analysed cohort…………………………... 12

Supplementary Figure 1. STROBE checklist………………………………………… 13-15

Supplementary Figure 2. Flow chart of examined patients………………………………… 16

Supplementary Figure 3. Number of subtype patients. 1-year mortality rate, symptomatic rate and infection rates by fluid collection subtype and enzyme kinetics..……………………….… 16

Supplementary Table 1. Further contributors of the Hungarian Pancreatic Study Group

Contributor	Centre	CRediT Authorship Contributions
**Andrea Harnos**	Centre for Translational Medicine, Semmelweis University, Budapest, Hungary<br><br>Department of Biostatistics, University of Veterinary Medicine, Budapest, Hungary	evaluation of the results
**Nelli Farkas**	Institute for Translational Medicine, Medical School, University of Pécs, Pécs, Hungary<br><br>Institute of Bioanalysis, Medical School, University of Pécs, Pécs, Hungary	
**Róbert Reszkető**	Institute of Pancreatic Diseases, Semmelweis University, Budapest, Hungary	patient inclusion administration
**Klaudia Káplár**	Institute of Pancreatic Diseases, Semmelweis University, Budapest, Hungary	
**Alexandra Mikó**	Institute for Translational Medicine, Medical School, University of Pécs, Pécs, Hungary<br><br>Department of Medical Genetics, Medical School, University of Pécs, Pécs, Hungary	
**Andrea Párniczky**	Institute for Translational Medicine, Medical School, University of Pécs, Pécs, Hungary<br><br>Heim Pál National Pediatric Institute, Budapest, Hungary	
**Balázs Csaba Németh**	Center for Gastroenterology, Department of Medicine, Albert Szent-Györgyi Medical School, University of Szeged, Szeged, Hungary<br><br>Hungarian Centre of Excellence for Molecular Medicine-University of Szeged, Translational Pancreatology Research Group, University of Szeged	interdisciplinary evaluation of the acute pancreatitis cases
**Balázs Kui**	Center for Gastroenterology, Department of Medicine, Albert Szent-Györgyi Medical School, University of Szeged, Szeged, Hungary	
**Szilárd Váncsa**	Centre for Translational Medicine, Semmelweis University, Budapest, Hungary	

**Rita Nagy**	Centre for Translational Medicine, Semmelweis University, Budapest, Hungary	
**Brigitta Teutsch**	Centre for Translational Medicine, Semmelweis University, Budapest, Hungary	
**Mahmoud Obeidat**	Centre for Translational Medicine, Semmelweis University, Budapest, Hungary	
**Jimin Lee**	Centre for Translational Medicine, Semmelweis University, Budapest, Hungary	
**Pál Ákos Deák**	Department of Interventional Radiology, Semmelweis University, Budapest, Hungary	
**Pál Maurovics-Horvát**	Medical Imaging Centre, Department of Radiology, Semmelweis University, Budapest, Hungary	
**Ibolya Kocsis**	Institute of Laboratory Medicine, Semmelweis University, Budapest, Hungary	
**Barna Vásárhelyi**	Institute of Laboratory Medicine, Semmelweis University, Budapest, Hungary	
**László Zubek**	Department of Anesthesiology and Intensive Therapy, Semmelweis University, Budapest, Hungary	
**Zsolt Molnár**	Department of Anesthesiology and Intensive Therapy, Semmelweis University, Budapest, Hungary	
**Dénes Horváthy**	Department of Interventional Radiology, Semmelweis University, Budapest, Hungary	
**László Gajdán**	Szent György Teaching Hospital of Fejér County, Székesfehérvár, Hungary	patient inclusion, data collection and data quality assurance
**Márta Varga**	Békés County Central Hospital, Dr. Réthy Pál Hospital, 2nd Ward of Gastroenterology, Békéscsaba, Hungary	
**Árpád Patai**	Markusovszky University Teaching Hospital, Szombathely, Hungary	
**József Hamvas**	Péterfy Street Hospital, Budapest, Hungary	
**Barnabás Bod**	Dr. Bugyi István Hospital, Szentes, Hungary	

**János Novák**	Pándy Kálmán Hospital of Békés County, Gyula, Hungary	
**Veronika Lillik**	Centre for Translational Medicine, Semmelweis University, Budapest, Hungary<br><br>Szent György Teaching Hospital of Fejér County, Székesfehérvár, Hungary	
**Katalin Márta**	Institute of Pancreatic Diseases, Semmelweis University, Budapest, Hungary	
**Dorottya Tarján**	Institute of Pancreatic Diseases, Semmelweis University, Budapest, Hungary	
**Zoltán Hajnády**	Institute of Pancreatic Diseases, Semmelweis University, Budapest, Hungary	
**Olga Julia Zahariev**	Institute of Pancreatic Diseases, Semmelweis University, Budapest, Hungary	
**Luca Havelda**	Institute of Pancreatic Diseases, Semmelweis University, Budapest, Hungary	
**Tamás Hussein**	Institute of Pancreatic Diseases, Semmelweis University, Budapest, Hungary	
**Péter Sahin**	Institute of Pancreatic Diseases, Semmelweis University, Budapest, Hungary	
**Tamás Tornai**	Institute of Pancreatic Diseases, Semmelweis University, Budapest, Hungary	
**Mónika Lipp**	Institute of Pancreatic Diseases, Semmelweis University, Budapest, Hungary	
**Emese Fürst**	Institute of Pancreatic Diseases, Semmelweis University, Budapest, Hungary	
**Edina Tari**	Centre for Translational Medicine, Semmelweis University, Budapest, Hungary<br><br>Szent György Teaching Hospital of Fejér County, Székesfehérvár, Hungary	
**Orsolya Eperjesi**	Institute of Pancreatic Diseases, Semmelweis University, Budapest, Hungary	

**Zoltán Bánfalvy**	Institute of Pancreatic Diseases, Semmelweis University, Budapest, Hungary	
**Boglárka Barna**	Institute of Pancreatic Diseases, Semmelweis University, Budapest, Hungary	
**Tibor Fehér**	Centre for Translational Medicine, Semmelweis University, Budapest, Hungary<br>Institute of Pancreatic Diseases, Semmelweis University, Budapest, Hungary<br>Department of Developmental Psychology, Institute of Psychology, Károli Gáspár University of the Reformed Church in Hungary	
**Zsófia Németh**	Institute of Pancreatic Diseases, Semmelweis University, Budapest, Hungary	
**Stefania Bunduc**	Centre for Translational Medicine, Semmelweis University, Budapest, Hungary<br><br>Digestive Disease and Liver Transplant Center, Fundeni Clinical Institute, Bucharest, Romania<br><br>Carol Davila University of Medicine and Pharmacy, Bucharest, Romania	

Supplementary Table 2. Centre distribution of the analysed cohort (22 Hungarian Centres)

**Centres**	**Patient number**
First Department of Medicine, University of Pécs, Pécs, Hungary	1186
Department of Medicine, University of Szeged, Szeged, Hungary	894
Szent György University Teaching Hospital of Fejér County, Székesfehérvár, Hungary	681
Institute of Pancreatic Diseases, Semmelweis University, Budapest Hungary	330
Bajcsy-Zsilinszky Hospital, Budapest, Hungary	158
Department of Internal Medicine, University of Debrecen, Debrecen, Hungary	144
Dr. Bugyi István Hospital of Csongrád County, Szentes, Hungary	81
BMKK Dr Réthy Pál Hospital, Békéscsaba, Hungary	75
Second Department of Medicine, Markusovszky University Teaching Hospital, Szombathely, Hungary	32
Pándy Kálmán Hospital of Békés County, Gyula, Hungary	28
Heim Pál Children's Hospital, Budapest, Hungary	21
Borsod-Abaúj-Zemplén County Hospital and University Teaching Hospital, Miskolc, Hungary	13
Csongrád County Hospital, Makó, Hungary	11
Bács-Kiskun County Hospital, Kecskemét, Hungary	10
Department of Pediatrics, Semmelweis University, Budapest, Hungary	12
Buda Hospital of the Order of Mercy	7
Balassa János Hospital of Tolna County, Szekszárd, Hungary	4
Bethesda Children's Hospital, Budapest, Hungary	2
Central Hospital of Northern Pest-Military Hospital, Budapest, Hungary	2
Uzsoki Street Hospital, Budapest, Hungary	1
Jósa András University Teaching Hospital of Szabolcs-Szatmár-Bereg County, Nyíregyháza, Hungary	1
Department of Pediatrics, University of Szeged, Szeged, Hungary	1
**All patients**	**3694**

6 **Supplementary Table 3.** Characteristics of the NEK and PPEE groups. IQR: interquartile range, BMI: body mass index, ERCP: endoscopic retrograde cholangiopancreatography, LOH: length of hospitalization, ICU: intensive care unit

	**Local complication group**	**A: Normal enzyme kinetics group (n=503)**	**B: Persistent pancreatic enzyme elevation group (n=276)**	**p-value (A->B)**
**Number of cases**	**912**	**503**	**276**	
**Sex**				
Male (%)	614 (67,32%)	334 (66.40%)	188 (68.12%)	0.64
Female (%)	298 (32,68%)	169 (33.60%)	88 (31.88%)	
**Age - years (median, IQR)**	55 (43-67)	58 (45-70)	52 (42-63,75)	**<0.001**
**BMI (median, IQR)**	27,54 (24,13-31,64)	28,71 (25.44-32.66)	25,73 (22.27-29.49)	**<0.001**
**Risk factors**				
Alcohol comsumption	494/892 (55,38%)	270/494 (54.66%)	155/271 (57.20%)	0.5
Smoking previously	476/886 (53,72%)	233/491 (47.45%)	165/269 (61.34%)	**<0.001**
**Etiology**				
Hypertrigliceridaemia	66/912 (7,24%)	43/503 (8.55%)	15/276 (5.43%)	0.11
Biliary	213/912 (23,36%)	140/503 (27.83%)	48/276 (17.39%)	**<0.001**
Alcoholic	230/912 (25,22%)	102/503 (20.28%)	86/276 (31.16%)	**0.001**
Idiopathic	179/912 (19,63%)	102/503 (20.28%)	56/276 (20.29%)	0.99
Post-ERCP	20/912 (2,19%)	15/503 (2.98%)	5/276 (1.81%)	0.29
Alcoholic+HTG	39/912 (4,28%)	26/503 (5.17%)	9/276 (3.26%)	0.2
Mixed	29/912 (3,18%)	18/503 (3.58%)	6/276 (2.17%)	0.29
Other	136/912 (14,91%)	57/503 (11.33%)	51/276 (18.48%)	**0.006**
**Severity**				
Mild	0/909 (0%)	0/502 (0%)	0/274 (0%)	0.98
Moderate	756/909 (83,17%)	418/502 (83.27%)	228/274 (83.21%)	
Severe	153/909 (16,83%)	84/502 (16.73%)	46/274 (16.79%)	
**Mortality 1-year follow-up**	118/754 (15,65%)	57/418 (13.63%)	33/228 (14.47%)	0.77
**In-hospital mortality**	67/906 (7,4%)	30/502 (5.98%)	17/273 (6.23%)	0.89
**LOH (mean, SD)**	13 (8-19)	17,62 (13.21)	19,25 (15.42)	0.11
**ICU needed at admission**	71/826 (8,6%)	42/467 (8.99%)	17/243 (7%)	0.36
**Antibiotic usage**	682/873 (78,12%)	418/487 (85.83%)	205/266 (77.07%)	**0.0024**
**Previous pancreatic disease**	291/841 (34,6%)	136/467 (29.12%)	105/251 (41.83%)	**<0.001**
**Organ failure during hospital stay**	217/911 (23,82%)	121/502 (24.10%)	62/276 (22.46%)	0.61

7 **Supplementary Table 4.** Factors influencing outcomes; Lower figure: severity by age and BMI (red: moderately severe, blue: severe)

		**Severity**		
	**OR**	**LCL**	**UCL**	**P.value**
Age	1.04	1.02	1.05	**0.0000**
Sex (male)	1.37	0.85	2.20	0.1956
PPEE	1.14	0.71	1.83	0.5989
BMI	1.06	1.02	1.10	**0.0021**
		**1-year mortality**		
	**OR**	**LCL**	**UCL**	**P.value**
Age	1.05	1.03	1.07	**0.0000**
Sex (male)	1.86	1.06	3.28	**0.0307**
PPEE	1.38	0.82	2.32	0.2195
Previous pancreatic diseases	0.54	0.30	0.98	**0.0428**
		**ICU admission**		
	**OR**	**LCL**	**UCL**	**P.value**
Age	1.02	1	1.04	0.1045
Sex (male)	0.97	0.54	1.75	0.9107
PPEE	0.83	0.45	1.51	0.5352
		**Organ failure**		
	**OR**	**LCL**	**UCL**	**P.value**
Age	1.01	1	1.03	0.0909
Sex (male)	1.2	0.69	2.07	0.5221
PPEE	0.72	0.41	1.26	0.2524
Previous pancreatic diseases	1.73	1.04	2.87	**0.0341**
		**Infected fluid collection**		
	**OR**	**LCL**	**UCL**	**P.value**
Age	0.95	0.92	0.99	**0.0066**
Sex (male)	1.29	0.79	2.12	0.3119
PPEE	1.86	1.15	3	**0.0114**

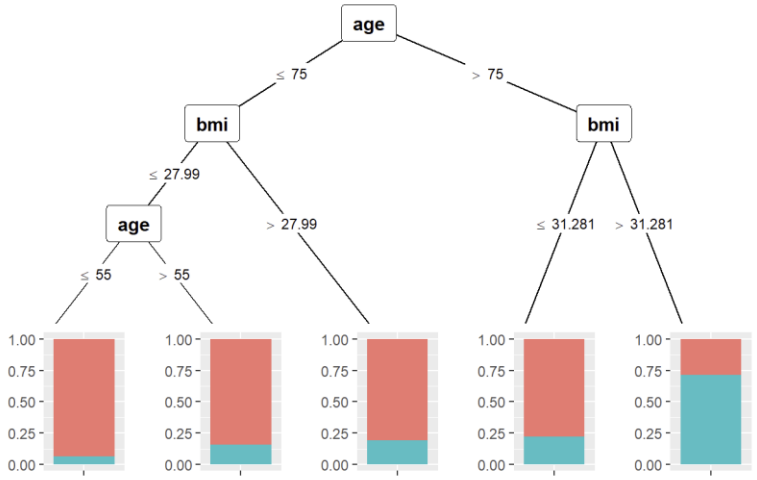
age
≤ 75
> 75
bmi
bmi
≤ 27.99
> 27.99
age
≤ 55
> 55
≤ 31.281
> 31.281
1.00
0.75
0.50
0.25
0.00

8 **Supplementary Table 5.** Data quality of the Hungarian Acute Pancreatitis Registry

**Variable**	**Total cohort**	**Uploaded data**	**%**
Age	3 694	3 694	**100.00%**
Sex	3 694	3 694	**100.00%**
BMI	3 694	3 132	**84.79%**
Etiology	3 694	3 694	**100.00%**
Severity (mild/moderate/severe)	3 694	3 683	**99.70%**
Mortality	3 694	3 004	**81.32%**
Local complications	3 694	3 694	**100.00%**
Smoking status	3 694	3 565	**96.50%**
Alcohol consumption status	3 694	3 582	**96.97%**
Amylase	3 694	2 445	**66.19%**
Lipase	3 694	1 787	**48.38%**
LOH	3 694	3 694	**100.00%**
ICU needed	3 694	3 350	**90.69%**
Antibiotics usage	3 694	3 522	**95.34%**
Previous pancreatitis	3 694	3 514	**95.13%**
Organ failure	3 694	3 689	**99.86%**
**Overall**	**59 104**	**53 743**	**90.93%**

9 **Supplementary Table 6.** Representativity of the analysed cohort

	Total cohort	Analyzed cohort
**Number of cases**	3,797	3,694
**Sex**		
Male (%)	2,157 (56.81%)	2,103 (56.93%)
Female (%)	1,640 (43.19%)	1,591 (43.07%)
**Age - years (median, IQR)**	58 (44-71)	58 (44-71)
**BMI (median, IQR)**	27.17 (23.66-31.48)	27.18 (23.67-31.48)
**Risk factors**		
Alcohol	1,742/3,677 (47.38%)	1,701/3,582 (47.49%)
Smoking previously	1,767/3,662 (48.25%)	1,723/3,565(48.33%)
**Etiology**		
Hypertrigliceridemia	140/3,797 (3.69%)	139/3,694 (3.76%)
Biliary	1,481/3,797 (39.00%)	1,462/3,694 (39.58%)
Alcoholic	683/3,797 (17.99%)	665/3,694 (18.00%)
Idiopathic	709/3,797 (18.67%)	669/3,694 (18.11%)
Post-ERCP	120/3,797(3.16%)	118/3,694 (3.19%)
Alcoholic+HTG	76/3,797 (2.00%)	73/3,694 (1.98%)
Mixed	77/3,797 (2.03%)	75/3,694 (2.03%)
Other	511/3,797 (13.46%)	493/3,694 (13.35%)
**Severity**		
Mild	2,561/3,777 (67.81%)	2,503/3,683 (67.96%)
Moderate	953/3,777 (25.23%)	927/3,683 (25.17%)
Severe	263/3,777 (6.96%)	253/3,683 (6.87%)
**Mortality 1 year follow up**	285/3,088 (9.23%)	275/3,004 (9.15%)
**In-hospital mortality**	113/3,704 (3.05%)	110/3,660 (3.01%)
**LOH (median, IQR)**	8 (6-12)	8 (6-12)
**ICU needed**	154/3,440 (4.48%)	151/3,350 (4.51%)
**Antibiotic usage**	2,344/3,617 (64.80%)	2,282/3,522 (64.79%)
**Previous pancreatic disease**	1,163/3,609 (32.22%)	1,121/3,514 (31.90%)
**Organ failure**	429/3,706 (11.58%)	424/3,689 (11.49%)

**Title and abstract**	1	(*a*) Indicate the study's design with a commonly used term in the title or the abstract	√
		(*b*) Provide in the abstract an informative and balanced summary of what was done and what was found	√
**Introduction**			
Background/rationale	2	Explain the scientific background and rationale for the investigation being reported	√
Objectives	3	State specific objectives, including any prespecified hypotheses	√
**Methods**			
Study design	4	Present key elements of study design early in the paper	√
Setting	5	Describe the setting, locations, and relevant dates, including periods of recruitment, exposure, follow-up, and data collection	√
Participants	6	(*a*) *Cohort study*—Give the eligibility criteria, and the sources and methods of selection of participants. Describe methods of follow-up<br>*Case-control study*—Give the eligibility criteria, and the sources and methods of case ascertainment and control selection. Give the rationale for the choice of cases and controls<br>*Cross-sectional study*—Give the eligibility criteria, and the sources and methods of selection of participants	√
		(*b*) *Cohort study*—For matched studies, give matching criteria and number of exposed and unexposed<br>*Case-control study*—For matched studies, give matching criteria and the number of controls per case	√
Variables	7	Clearly define all outcomes, exposures, predictors, potential confounders, and effect modifiers. Give diagnostic criteria, if applicable	√
Data sources/ measurement	8*	For each variable of interest, give sources of data and details of methods of assessment (measurement). Describe comparability of assessment methods if there is more than one group	√
Bias	9	Describe any efforts to address potential sources of bias	√
Study size	10	Explain how the study size was arrived at	√
Quantitative variables	11	Explain how quantitative variables were handled in the analyses. If applicable, describe which groupings were chosen and why	√
Statistical methods	12	(*a*) Describe all statistical methods, including those used to control for confounding	√
		(*b*) Describe any methods used to examine subgroups and interactions	√

		(*c*) Explain how missing data were addressed	√
		(*d*) *Cohort study*—If applicable, explain how loss to follow-up was addressed<br>*Case-control study*—If applicable, explain how matching of cases and controls was addressed<br>*Cross-sectional study*—If applicable, describe analytical methods taking account of sampling strategy	√
		(*e*) Describe any sensitivity analyses	√
**Results**			
Participants	13*	(a) Report numbers of individuals at each stage of study—eg numbers potentially eligible, examined for eligibility, confirmed eligible, included in the study, completing follow-up, and analysed	√
		(b) Give reasons for non-participation at each stage	√
		(c) Consider use of a flow diagram	√
Descriptive data	14*	(a) Give characteristics of study participants (eg demographic, clinical, social) and information on exposures and potential confounders	√
		(b) Indicate number of participants with missing data for each variable of interest	√
		(c) *Cohort study*—Summarise follow-up time (eg, average and total amount)	√
Outcome data	15*	*Cohort study*—Report numbers of outcome events or summary measures over time	√
		Case-control study—Report numbers in each exposure category, or summary measures of exposure	
		Cross-sectional study—Report numbers of outcome events or summary measures	
Main results	16	(*a*) Give unadjusted estimates and, if applicable, confounder-adjusted estimates and their precision (eg, 95% confidence interval). Make clear which confounders were adjusted for and why they were included	√
		(*b*) Report category boundaries when continuous variables were categorized	√
		(*c*) If relevant, consider translating estimates of relative risk into absolute risk for a meaningful time period	√
Other analyses	17	Report other analyses done—eg analyses of subgroups and interactions, and sensitivity analyses	√
**Discussion**			
Key results	18	Summarise key results with reference to study objectives	√
Limitations	19	Discuss limitations of the study, taking into account sources of potential bias or imprecision. Discuss both direction and magnitude of any potential bias	√
Interpretation	20	Give a cautious overall interpretation of results considering objectives, limitations, multiplicity of analyses, results from similar studies, and other relevant evidence	√

Generalisability	21	Discuss the generalisability (external validity) of the study results	√
**Other information**			
Funding	22	Give the source of funding and the role of the funders for the present study and, if applicable, for the original study on which the present article is based	√

10 Supplementary Figure 1. STROBE checklist

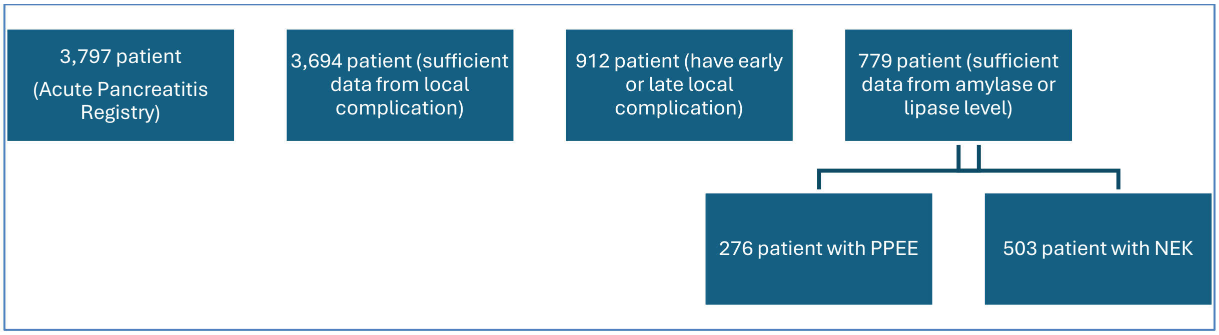

11 **Supplementary Figure 2. Flow chart of examined patients**

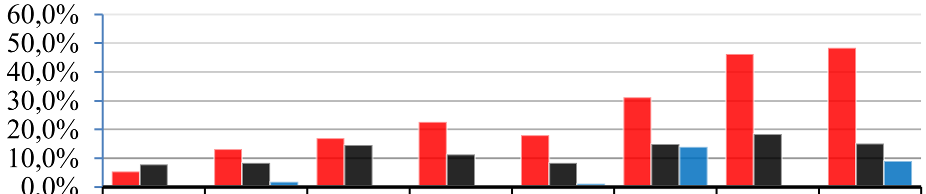

	APFC - NEK (202)	APFC - PPEE (106)	ANC - NEK (164)	ANC - PPEE (44)	Pseudocyst - NEK (83)	Pseudocyst - PPEE (93)	WON - NEK (54)	WON - PPEE (33)
Pancreatic fluid infection rate	5,4%	13,2%	17,1%	22,7%	18,1%	31,2%	46,3%	48,5%
Mortality rate - 1 year	7,9%	8,5%	14,6%	11,4%	8,4%	15,1%	18,5%	15,2%
Symptomatic rate	0,0%	1,9%	0,0%	0,0%	1,2%	14,0%	0,0%	9,1%

Pancreatic fluid infection rate   Mortality rate - 1 year   Symptomatic rate

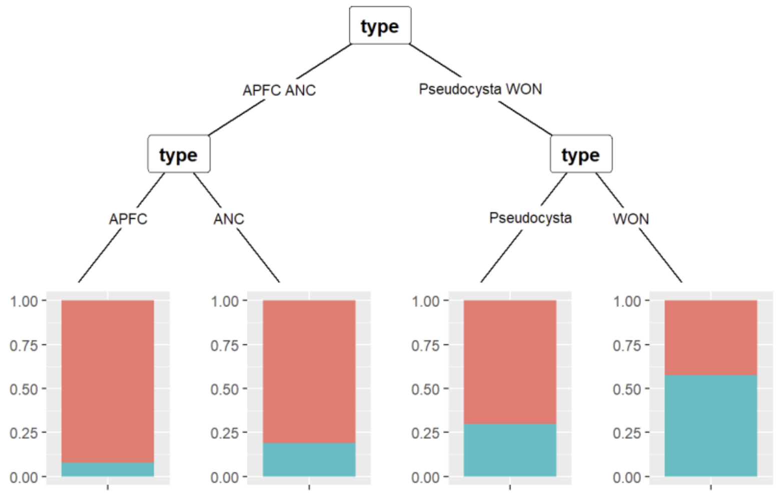

12 **Supplementary Figure 3.** Upper: Number of subtype patients. 1-year mortality rate, symptomatic rate and infection rates by fluid collection subtype and enzyme kinetics, Lower: Infection rate by revised Atlanta classification without known pancreatic enzyme levels (n:912) (red: no superinfection of fluid collections, blue: fluid collections with superinfection)
